# Toward an Interdisciplinary Understanding of Sensory Dysfunction in Autism Spectrum Disorder: An Integration of the Neural and Symptom Literatures

**DOI:** 10.3389/fnins.2016.00268

**Published:** 2016-06-17

**Authors:** Kimberly B. Schauder, Loisa Bennetto

**Affiliations:** Department of Clinical and Social Sciences in Psychology, University of RochesterRochester, NY, USA

**Keywords:** sensory processing, autism spectrum disorder, interdisciplinary approaches, sensory symptoms, hyper-responsiveness, hypo-responsiveness

## Abstract

Sensory processing differences have long been associated with autism spectrum disorder (ASD), and they have recently been added to the diagnostic criteria for the disorder. The focus on sensory processing in ASD research has increased substantially in the last decade. This research has been approached from two different perspectives: the first focuses on characterizing the symptoms that manifest in response to real world sensory stimulation, and the second focuses on the neural pathways and mechanisms underlying sensory processing. The purpose of this paper is to integrate the empirical literature on sensory processing in ASD from the last decade, including both studies characterizing sensory symptoms and those that investigate neural response to sensory stimuli. We begin with a discussion of definitions to clarify some of the inconsistencies in terminology that currently exist in the field. Next, the sensory symptoms literature is reviewed with a particular focus on developmental considerations and the relationship of sensory symptoms to other core features of the disorder. Then, the neuroscience literature is reviewed with a focus on methodological approaches and specific sensory modalities. Currently, these sensory symptoms and neuroscience perspectives are largely developing independently from each other leading to multiple, but separate, theories and methods, thus creating a *multidisciplinary* approach to sensory processing in ASD. In order to progress our understanding of sensory processing in ASD, it is now critical to integrate these two research perspectives and move toward an *interdisciplinary* approach. This will inevitably aid in a better understanding of the underlying biological basis of these symptoms and help realize the translational value through its application to early identification and treatment. The review ends with specific recommendations for future research to help bridge these two research perspectives in order to advance our understanding of sensory processing in ASD.

## Introduction

Autism spectrum disorder (ASD) is characterized by deficits in social communication and the presence of restricted and repetitive behaviors, including sensory atypicalities. Sensory processing abnormalities have been reported in ASD since the earliest firsthand and clinical accounts (Kanner, 1943; Cesaroni and Garber, 1991). Recent estimates suggest a high prevalence of sensory symptoms, with reports ranging from 60 to 96% of children with ASD exhibiting some degree of atypical responses to sensory stimuli (Dunn et al., 2002; Baranek et al., 2006; Billstedt et al., 2007; Leekam et al., 2007; Klintwall et al., 2011; Lane et al., 2011; Marco et al., 2011). The updated diagnostic criteria for ASD in DSM-5 includes abnormal sensory behaviors (American Psychological Association, 2013). Sensory processing differences likely contribute to many of the higher-order cognitive and social deficits associated with ASD (Cascio et al., 2016), highlighting the broad potential impact of atypical sensory processing. Understanding the mechanisms through which these sensory symptoms manifest could help parents, educators, clinicians, and individuals themselves attend to the sensory environment and make adjustments accordingly in the hopes of normalizing one's sensory experiences and alleviating any downstream effects of atypical responding to sensory input. Over the last decade, there has been a significant increase in research related to sensory processing in ASD, which builds upon the initial accounts and research studies in this domain (see Rogers and Ozonoff, 2005, for a review). This accumulating research has been approached from two major perspectives. The first focuses on the symptoms that manifest in response to real world sensory stimulation. The second focuses on the neural pathways and mechanisms underlying sensory processing.

Currently, the symptom literature largely utilizes self- or parent- report questionnaires and/or observational, lab-based paradigms, in an attempt to characterize the observable reactions that impact an individual on a daily basis. Current neuroscience approaches measure the degree and timing of neural response. Despite each field lending itself to the study of unique aspects of sensory processing in ASD, there is a gap in our understanding of how neural response to sensory input is related to symptoms. Nonetheless, neural reactivity and processing patterns underlie and give rise to the presence of sensory symptoms (Marco et al., 2011), making it essential to understand *how* neural processing contributes to sensory symptoms. Each field has unique strengths, which have led to important contributions in our understanding of sensory processing in ASD; however, they each have a lot to learn from the other as we begin to bridge these two fields to gain a more comprehensive picture of this newly recognized diagnostic feature of ASD.

The purpose of this paper is to integrate the current research perspectives and methodological approaches related to sensory processing in ASD. Specifically, we will first clarify important terminology that is currently used in the study of sensory processing in ASD. Then, empirical studies from the last decade that focus on non-social, unisensory experiences, including those from both the sensory symptoms and neuroscience perspectives, will be reviewed with the goal of increased understanding of each respective field in order to move toward an interdisciplinary approach to sensory processing in ASD. Some recent studies have begun to integrate these perspectives (Green et al., 2013, 2015; Cascio et al., 2015) and provide a basis to further build upon. Furthermore, both of these perspectives investigate various domains of ASD (e.g., sensory, cognitive, social, language), and ASD research as a whole would benefit from integration of these perspectives. However, given that sensory processing differences potentially have broad downstream effects in higher-order domains (e.g., cognitive, social, language), it is essential to first bridge these perspectives at the sensory level. Thus, although a systematic review of all pieces of sensory processing in ASD is beyond the scope of this paper, its goal is to provide a foundation for shared understanding among disciplines investigating sensory processing in ASD.

### Terminology

In order to integrate the literatures on sensory processing in ASD, it is important to clarify the current terminology used across fields. Recently, Cascio et al. (2016) highlighted the inconsistent conceptualizations and definitions across fields related to sensory processing in ASD and how this poses a challenge to cross-discipline communication and collaboration. The definitions that follow are an attempt to summarize and organize the existing terms used to describe sensory processing in ASD (Table 1). Our goal is to highlight the many components of sensory processing that might exist and to highlight the nuanced differences between these possible components of sensory processing.

**Table 1:** **Terminology in sensory processing in ASD research**.

**Term**	**Definition in symptom research**	**Definition in neuroscience research**
Sensory processing	The process of the brain registering sensory input from the outside world and the individual generating a response based on that input	
Symptom	Atypical responses to sensory input that interfere with an individual's daily functioning	
Behavior	Observable reactions	Ability to detect or discriminate, measured by perceptual decisions
Low threshold	Requires less sensory input to generate a typical response (referred to as low threshold to stimulation)	Requires less sensory input to generate a neural response
High threshold	Requires more sensory input to generate a typical response (referred to as high threshold to stimulation)	Requires more sensory input to generate a neural response
Hyper-responsiveness	Presence of an atypical response, or over-reaction	Increased neural responding
Hypo-responsiveness	Absence of a typical response, or under-reaction	Decreased neural responding
Sensory gating		Inhibitory functioning at the neural level, which filters out redundant or unnecessary neural responses to all other environmental stimuli
Sensitivity	Negative reaction to sensory input	Degree to which one is susceptible to perceiving small changes in stimulus intensity; inverse of perceptual threshold
Habituation	Decreased response of the individual to repetitive sensory stimulation	Decreased neural response to repetitive sensory stimulation
Defensiveness	Negative reaction to sensory input	
Avoidance	Resistance or unwillingness to interact with sensory stimuli	
Poor registration	Decreased ability to register sensory input (typically measured by lack of response)	
Sensory orienting	Directed attention to a sensory stimulus	
Sensory Filtering	Ability to process relevant sensory information and exclude irrelevant/distracting sensory information	
Sensory Seeking	Excessively seeking out sensory input	

*These definitions are adopted from the literature, but they are fine-tuned to emphasize important differences between terms that are oftentimes used interchangeably*.

Terminology confusion exists for two main reasons. First, both the neural and symptoms literatures adopt some of the same terms, but these terms oftentimes refer to very different concepts. For example, *behavior* is a problematic term because it is conceptualized differently across fields. Namely, neuroscientists discuss behavior in terms of perceptual decisions (e.g., detection or discrimination abilities; Weigelt et al., 2012), whereas clinicians tend to focus on observable reactions (e.g., a child covering his/her ears at the sound of a blender; McCormick et al., 2016). The second point of confusion regarding terminology surrounds the large range of terms within the sensory symptom literature that are oftentimes used interchangeably, but are arguably different constructs. The discussion that follows highlights both aspects of this confusion.

**Figure 1:**
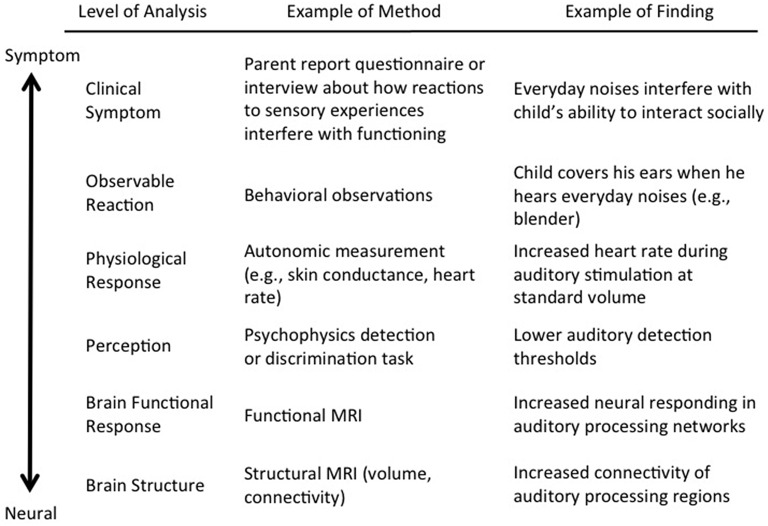
**Conceptual map of sensory processing using a common example of hyper-responsiveness to auditory stimulation**. The first column outlines various levels of analysis from brain to symptom. For the sake of simplicity, these are organized using a bottom-up conceptualization in a single pathway from brain to symptom; however, in reality, there are likely several different pathways from brain to symptom, including bidirectional relationships between certain levels. The second column provides an example of an appropriate method for each level of analysis. The third column provides one example of a possible finding related to auditory hyper-responsiveness using each particular method.

*Sensory processing* is the umbrella term that refers to the entire process from the brain registering sensory input from the outside world to the individual generating a response based on that input. Atypical neural responding to sensory input is thought to impact responses at multiple levels, including perceptual, physiological, and observable differences (Marco et al., 2011; Figure 1). Clinically, atypical sensory processing is manifested in inappropriate responses to sensory input that involve emotional and behavioral disruptions, and interfere with an individual's daily functioning (Miller et al., 2007). More generally, these disruptions can be considered *sensory symptoms*. Although it is understood that sensory symptoms are a result of how sensory information is processed in the brain and the body (Cascio et al., 2016), the translation from neural firing to sensory symptoms is a complex process.

Early models of sensory processing, developed to explain behavior and plan clinical interventions, emphasized both neurological thresholds and response patterns in the generation of sensory reactions, or symptoms (Ayres, 1972; Dunn, 1997). Neurological thresholds refer to the amount of sensory input needed for the brain to register that input. Clinically, *low threshold to stimulation* is thus conceptualized as an individual requiring less sensory input to generate the typical response, whereas *high threshold to stimulation* is conceptualized as an individual requiring more sensory input to generate a typical response. These thresholds are important because it follows that typical levels of sensory input might then generate an atypical response—over-reaction for low threshold to stimulation and under-reaction for high threshold to stimulation. Researchers who have adopted these models generally rely on questionnaires to measure these constructs (e.g., Joosten and Bundy, 2010; Reynolds et al., 2011). However, questionnaires are unable to accurately measure an individual's threshold to stimulation, and thus rely on an assumption that an observable reaction accurately captures the complexity of processing sensory input (see Sensory Symptoms section for more information).

Neuroscientists measure this construct of neurological threshold more directly, but have adopted different terminology. At the neural level, *hyper-* and *hypo-responsiveness*^1^ refer to increased or decreased neural firing, respectively (e.g., Gomot et al., 2008). Additionally, thresholds are determined by the process of sensory gating and habituation. *Sensory gating* refers to the brain's inhibitory ability to filter out redundant or unnecessary neural responses to irrelevant environmental stimuli (Orekhova et al., 2008). *Habituation* refers to decreased neural response to repetitive sensory stimulation (e.g., Guiraud et al., 2011). At the perceptual level, *sensitivity* is determined by the smallest stimulus intensity that is detectable, and is defined as the inverse of a perceptual threshold (Engen, 1988). Adding to the confusion, sensory symptom researchers define *sensitivity* as a negative reaction in response to low threshold to stimulation (Dunn, 1997). In addition, they refer to *hyper-responsiveness* as the presence of an atypical response, such as covering one's ears in response to everyday noises, and *hypo-responsiveness* as the absence of a typical response, such as failure to orient to salient sounds (e.g., Ben-Sasson et al., 2009; McCormick et al., 2016). Based on the theoretical conceptualization that these observable components are a result of neurological thresholds, hyper- and hypo-responsiveness have thus each become more of a single construct than is theoretically warranted. Furthermore, because of this parallel terminology, connections between neural firing patterns and symptoms are often portrayed as overly simplistic.

The complexity of sensory processing is further highlighted when incorporating the response pattern component, which suggests a wide range of possible symptoms that could result from atypical neural registration and perception of sensory stimuli. *Sensory defensiveness* is observed as a negative reaction to a sensory stimulus that is not typically considered to be aversive, such as repeatedly itching or screaming in reaction to a tag in clothing (Baranek et al., 1997). *Sensory avoidance* is observed as a resistance or unwillingness to interact with the stimulus (Dunn, 1997). *Poor registration* is defined as decreased ability to register sensory input and is oftentimes measured by a lack of response (Dunn, 1997). Conversely, *sensory orienting* is the direction of attention to a sensory stimulus, which is assumed to be in response to successful registration of that stimulus (Liss et al., 2006). Similar to the neuroscience definition, *habituation* is defined as decreased response to repetitive sensory stimulation; however, in this literature, it is measured by orienting responses, often to repeated sounds (Baranek et al., 2007). *Sensory filtering*, which has been predominately defined in the auditory modality, refers to the ability to selectively attend to relevant sensory information and exclude irrelevant or distracting sensory information (Tomchek and Dunn, 2007). *Sensory seeking* is defined as excessively seeking out sensory input (Dunn, 1997; Liss et al., 2006).

Although these symptoms are uniquely defined, these terms are sometimes being operationalized more broadly (e.g., defensiveness as high sensitivity or having a low threshold to stimulation; Kern et al., 2006), rather than focusing exclusively on the observable reaction. Furthermore, in some cases it is unclear as to how these symptoms originate from neural responding patterns. For example, sensory seeking was originally conceptualized as a compensatory response in an individual with high threshold to stimulation (Dunn, 1997), but has also been conceptualized as a compensatory response to overarousal (Liss et al., 2006), which would theoretically occur in individuals with low threshold to stimulation. This example underscores the importance of measuring both the neural response and the symptom presentation and cautions against assuming that the observable reaction accurately captures the complexity of sensory processing.

In sum, the conceptualization of sensory processing as a unitary construct is challenged by the broad array of existing terms that each define specific components of this complicated process. The inconsistent definitions across disciplines, highlighted above, have contributed to confusion within the study of sensory processing in ASD. At the neural level, thresholds determine which sensory information is registered in the brain. This then influences perceptual sensitivity and bodily response, and leads to observable reactions and symptoms (Figure 1). The literature defines and differentiates between several possible symptom presentations (defensiveness, avoidance, poor registration, poor habituation, poor filtering, seeking), measured mainly at the level of observable reactions. Organizing these terms and reconciling the manner in which they are used in both the neural and symptom literatures provides an important step toward clarifying the often overly simplified connections between sensory symptoms and their underlying neural patterns and promoting more collaborative future research.

## Sensory symptoms in ASD

In the past 10 years, sensory experiences of individuals with ASD have been assessed via three methods: self-, parent-, and teacher-report questionnaires; behavioral observations; and autonomic measurement. Questionnaire measures are by far the most commonly used, and have largely been successful in clinical settings to generally discriminate typical from atypical sensory processing. However, their ability to more sensitively capture individual differences in sensory processing is less clear for three main reasons. First, the inherent nature of questionnaires limits their utility to assessing components of sensory processing to those that can be observed. However, many of the sensory questionnaire measures yield scores that imply information about other components of sensory processing. For example, Poor Registration, a factor score on the commonly used Sensory Profile (Dunn, 1999), includes items such as “decreased awareness of pain and temperature,” “doesn't notice when people come into the room,” and “does not seem to smell strong odors.” Thus, all of these infer poor registration of the stimulus from an observation.

Secondly, a common practice in sensory questionnaire measures is to combine items that target different levels of sensory processing. For example, the Hyperresponsiveness score of the Sensory Experience Questionnaire (Baranek et al., 2006) includes items such as “does your child notice sounds in the environment before other people do?,” “is your child disturbed by too much light inside or brightness outside?,” “does your child react negatively or pull away when touched by a person?” Thus, this score collapses the perceptual, emotional, and observable responses across multiple sensory modalities, and possibly misses important differences that may occur within more precisely defined sensory processing levels and modalities. Finally, many sensory questionnaires include some items evaluating social-sensory experiences (e.g., does your child respond to his/her name?; Dunn, 1999; Baranek et al., 2006), making it impossible to disentangle the independent contributions of sensory and social components. Given these limitations of questionnaire measures in the assessment of sensory processing in ASD, the literature that follows mostly captures a coarse picture of the observable reactions component of sensory processing. To parallel this level of inquiry, we will use the general terms of hyper- and hypo-responsiveness in our discussions of findings and will clarify specific aspects of those responding patterns when appropriate.

In the past few years, there have been some positive advancements in the development and analysis of questionnaire measures that provide promising avenues for future research. Specifically, more focused questionnaires have been developed to address some of the limitations outlined above. An example is the Sensory Perception Quotient (Tavassoli et al., 2014a). This questionnaire focuses on basic detection and discrimination abilities and includes items that target specific sensory receptors across a variety of sensory modalities (e.g., “I find it difficult to see individual stars on a clear night” and “I would be the first to hear if there was a fly in the room”). Thus, this questionnaire is still limited to observable responses, but begins to selectively target the perceptual level of analysis. Another advancement that has recently been applied to sensory questionnaires is the use of cluster-based statistical analysis to identify sensory-based subtypes within ASD. Subtype identification is motivated by the known heterogeneity within ASD and has implications for neurobiological studies that aim to link sensory features with specific underlying mechanisms. Such studies have yielded somewhat inconsistent subtypes (Lane et al., 2010, 2011, 2014; Ausderau et al., 2014), which is possibly due to imprecise measurement tools. Nonetheless, this subtype identification approach is a positive step in our understanding of underlying mechanisms.

Other methods that assess sensory symptoms include lab-based observational paradigms and autonomic measurements to characterize physiological responses. Lab-based observational paradigms are similar to questionnaire measures in that they largely focus on observable reactions to real world sensory input (e.g., toys, objects with certain sensory features). However, they differ from questionnaire methods in that they rely on behavioral coding in the lab to obtain a more controlled and objective measure of these symptoms. Psychophysiological studies focus on the body's response to sensory stimulation, specifically looking at functioning of the autonomic nervous system (ANS). Thus, these studies provide objective measures of bodily states that have been linked with emotional states. The following sections review the sensory symptom literature that uses questionnaires, lab-based observational coding paradigms, and psychophysiological methodologies.

### Questionnaire-based studies

Questionnaire-based studies on sensory processing in ASD, detailed below, have culminated in two major conclusions: (1). Individuals with ASD respond to sensory input in ways different from the typical population, across a variety of modalities and across the entire lifespan, and (2). Sensory processing differences are related to a variety of the core and associated symptoms of ASD and affect the quality of life in these individuals.

#### Sensory symptoms across development

Some studies examined developmental trends in samples spanning large age ranges. These studies showed atypicalities in ASD across all sensory modalities that decrease with age (Kern et al., 2006; Leekam et al., 2007). Additionally, symptoms in different modalities (e.g., visual, auditory, tactile) were found to be moderately correlated with each other across the lifespan, but only correlated with general autism symptom severity in childhood. (Kern et al., 2007). These findings support a general disruption in sensory processing, with responses to stimuli in each modality being related to each other, at least at the symptom level. Additionally, these findings suggest a maturational process that leads to a decrease in sensory symptoms with increasing age that is independent from change in autism severity more globally. Given these general developmental trends, studies investigating narrower age ranges provide more detailed information at important developmental stages.

Four studies have specifically looked at the profile of sensory symptoms in infants and toddlers. Patterns of hyper-responsiveness best differentiated those with ASD from those with typical development (TD); this was seen both within the modalities of touch, audition, and taste/smell (Wiggins et al., 2009), and across low threshold patterns more globally including both sensitivity and avoidance (Ben-Sasson et al., 2007). These sensory symptoms presented irrespective of cognitive ability and expressive language level (Ben-Sasson et al., 2007; Klintwall et al., 2011). However, hypo-responsiveness best differentiated toddlers with ASD from those with developmental delay and TD, suggesting that a pattern of decreased responding may be the most unique pattern in ASD (Baranek et al., 2006), a finding confirmed by a meta-analytic review (Ben-Sasson et al., 2009). A subgroup of toddlers with ASD had atypical scores across multiple patterns, highlighting the comorbidity of these symptoms even at this early developmental stage (Baranek et al., 2006; Ben-Sasson et al., 2007). A study using high-risk infants found that those who developed ASD had more auditory symptoms and hypo-responsive patterns compared to high-risk infants that do not develop ASD and low-risk infants (Germani et al., 2014), suggesting that these may be risk factors for developing ASD and making these symptoms potentially important for early identification of the disorder.

In slightly older (3–6 years old) children, Tomchek and Dunn (2007) reported similar findings of global hypo-responsiveness as well as hyper-responsiveness in the auditory, tactile, and taste/smell modalities. Also in line with the findings in infants and toddlers, sensory symptoms in children do not seem to be related to cognitive abilities (O'Donnell et al., 2012). However, these sensory symptoms do seem to be related to language skills and adaptive functioning in children (Tomchek et al., 2015). McCormick et al. (2016) examined developmental trajectories of sensory symptoms in children with ASD compared to those with other developmental disorders and TD, and found that children with ASD have elevated sensory symptoms from an early age (2 years) that remain stable through the age of 8, and that hyper-responsiveness in the taste/smell modality and poor auditory filtering best differentiated ASD from other developmental disorders. In a large study of 3–10 year old children, sensory symptoms across modalities were higher in children with ASD and ADHD compared to those with TD (Cheung and Siu, 2009). Although the ASD and ADHD groups were not distinguishable overall, as age increased, the ASD group showed decreases in symptoms while the ADHD group showed stable or increased symptoms. Finally, two studies investigated sensory symptoms in the home vs. school environments, and demonstrated both shared and unique expression of sensory symptoms across contexts (Brown and Dunn, 2010; Fernandez-Andres et al., 2015).

In adolescents, two studies found less sensory seeking in ASD (De La Marche et al., 2012; Stewart et al., 2016) with De La Marche et al. (2012) also showing more hyper-responsiveness (specifically in sensory avoidance) and Stewart et al. (2016) also showing hypo-responsiveness (Low Registration). Using a small sample of adults, Crane et al. (2009) found similar self-reported symptoms in adults with ASD compared to TD adults, with the most obvious differences in sensory avoidance. These sensory scores were all correlated with IQ, suggesting that higher IQ in adults may serve as a protective factor against persistent sensory symptoms. Increased levels of self-reported hyper-responsiveness in a large sample of adults with ASD have been shown using the SensOR (Tavassoli et al., 2014b) and the Sensory Processing Quotient (Tavassoli et al., 2014a), measures specifically targeting hyper-responsive sensory symptoms.

Developmentally, the literature paints a picture of early hypo-responsiveness, as well as hyper-responsiveness particularly in the auditory, taste/smell, and touch modalities; these symptoms pervasively affect individuals with ASD and remain stable through at least 8 years of age. The pattern of sensory seeking remains unclear, and may be due in part to the varying conceptualizations of these symptoms; additional work is needed to characterize this pattern of symptoms. Although sensory symptoms appear to be unrelated to more global functioning in toddlers, sensory symptoms begin to show relationships with adaptive functioning and language skills in early childhood. As individuals with ASD mature, hyper-responsive symptoms best characterize adults. This could indicate that early hypo-responsiveness leads to later global hyper-responsiveness or alternatively, could be due to differences in parent- vs. self-reporting strategies that are typically used in younger children and adults, respectively. If the latter is the case, it is possible that subjective and observed experiences differ, raising the importance of collecting multiple types of data in the study of sensory symptoms in ASD.

#### Sensory symptoms and their relation to other ASD symptoms and challenges

Several studies have examined relationships between sensory differences and a variety of the core and associated symptoms of ASD, revealing important information about the functional impact of sensory processing and its interference in day-to-day life. Three studies have looked at the relationship between sensory symptoms and general autism symptoms. In school-aged children, general sensory symptoms measured by the Sensory Processing Measure were related to autism severity measured by the Gilliam Autism Rating Scale, 2nd Edition in both the home and school environment (Sanz-Cervera et al., 2015). In a similarly aged sample, Hilton et al. (2010) used the Sensory Profile and identified that more proximal senses (touch, taste) may be more related to social difficulties, measured by the Social Responsiveness Scale in children with ASD. In adults both with and without ASD, Tavassoli et al. (2014b) showed a relationship between self-reported sensory hyper-responsiveness and autism symptoms.

Other studies have looked at sensory processing in relation to more specific types of functioning, such as school-related difficulties and activity participation. In school-aged children with ASD, Ashburner et al. (2008) found empirical support for the theoretical links between atypical sensory responding and difficulties in classroom settings. Specifically, they found hypo-responsiveness and difficulties with auditory filtering to explain about half of the variance in academic performance, above and beyond IQ and general ASD symptoms. Additionally, tactile hyper-responsiveness and auditory filtering deficits explained 36% of the variance in inattention problems. Together, these findings show the impact of sensory symptoms on children's ability to pay attention and perform successfully in school. Different sensory responding profiles have also been linked to different preferred activities; children with sensory hyper-responsiveness participate less in social, school, and extracurricular activities (Reynolds et al., 2011; Little et al., 2015), while children with sensory hypo-responsiveness participate more in activities outside the home, which the authors speculate may be because they are more passive (Little et al., 2015). These sensory responding and functional impairment relationships also exist in younger children with ASD; preschoolers with more significant sensory abnormalities also have more behavior problems (O'Donnell et al., 2012).

Ben-Sasson et al. (2008) used cluster profiles in toddlers and found that those with high hyper- and hypo-responsiveness also had more negative emotions and higher levels of anxiety and depression symptoms. These authors speculated that internalizing disorders in ASD may develop from the accumulated negative experiences with sensory input throughout development. Sensory hyper-responsiveness, specifically, was moderately correlated with anxiety in a very large sample of children with ASD (Mazurek et al., 2013). Green et al. (2012) longitudinally tested the relationship between sensory hyper-responsiveness and anxiety in 149 toddlers with ASD. This study confirmed the correlation between sensory hyper-responsiveness and anxiety in ASD, and importantly established a directional link from sensory hyper-responsiveness to anxiety. While sensory hyper-responsiveness remained stable and predicted change in anxiety over time, anxiety levels increased over time and did not predict change in sensory hyper-responsiveness. These findings suggest that sensory hyper-responsiveness may emerge earlier than and exacerbate the presentation of anxiety, or that these symptoms may have a common cause with different developmental manifestations.

Sensory hyper-responsiveness has also been linked to gastrointestinal (GI) problems (Mazurek et al., 2013), picky eating (Cermak et al., 2010; Nadon et al., 2011), sleep problems (Mazurek and Petroski, 2015), more externalizing behaviors, and increased parenting stress and family impairment (Ben-Sasson et al., 2013). Mazurek et al. (2013) proposed that sensory hyper-responsiveness, anxiety, and GI problems may be explained by shared neural mechanisms through which heightened stress leads to physiological and cognitive symptoms of anxiety in addition to experiencing particular stimuli as aversive. Individuals exhibiting this trio of symptoms may represent a unique subgroup of ASD. Furthermore, sensory hyper-responsiveness has been linked with increased sleep problems through similar hypothesized mechanisms (Mazurek and Petroski, 2015). Overall, this body of work focusing specifically on sensory hyper-responsiveness represents theoretically relevant and convincing evidence for the link between sensory hyper-responsiveness and anxiety, GI, sleep, and other problems in ASD. The observable nature of hyper-responsive reactions likely contributes to the comprehensiveness and replicability of this finding.

Several studies have examined the relationship between sensory symptoms and restricted and repetitive behaviors. Among children 5–18 years with intellectual disability, hyper-responsiveness (sensory sensitivity and sensory avoidance) distinguished those with ASD from those without ASD (Joosten and Bundy, 2010). The authors suggest a mechanism whereby hyper-responsiveness leads to increased anxiety, which subsequently leads to engagement in low-level repetitive behaviors to cope with that anxiety. Boyd et al. (2009) investigated relationships between sensory responding (measured by the Sensory Questionnaire), repetitive behaviors (measured by the Repetitive Behavior Scale—Revised), and executive functioning (measured by the Behavior Rating Inventory of Executive Function) in 6–17 year olds with and without ASD. No hypothesized relationships were found between sensory responding and executive functioning; however, sensory responding was related to two specific types of repetitive behaviors: stereotypy and compulsions. This data did not support the theoretical claim that executive dysfunction is the shared mechanism for sensory symptoms and repetitive behaviors in ASD, and the authors suggest that neurobiological, rather than neurocognitive, mechanisms might better explain this link. In other words, it is possible that the questionnaire measure designed to target neurocognitive processes does not accurately capture the neurobiological underpinnings that give rise to these neurocognitive processes. Wigham et al. (2015) investigated the relationship between sensory symptoms, repetitive behaviors, and intolerance of uncertainty, finding that the relationship between sensory responding and repetitive behaviors (particularly sameness behaviors) was mediated by intolerance of uncertainty, providing a specific cognitive explanation for the relationship. These studies provide some initial empirical evidence to support the conceptual relationship between sensory processing and repetitive behaviors in ASD, highlighting intolerance of uncertainty, but not executive functioning, as a possible mediating factor.

In sum, questionnaire-based studies have repeatedly confirmed that sensory processing is atypical in ASD at the level of reported observable reactions. Specific aspects of sensory processing difficulties, including hyper-responsiveness in the modalities of touch, audition, and taste/smell and patterns of general hypo-responsiveness, have most consistently emerged in the questionnaire-based literature with other differences being less consistently reported. Thus, patterns of both hyper- and hypo- responsiveness in ASD exist at the group level, with hyper-responsiveness further broken down at the level of sensory modality. This may reflect greater differentiation of sensory processing abilities in the hyper-responsiveness, vs. hypo-responsiveness, pattern, but it likely reflects the better precision of characterizing these symptoms given that they are defined by the *presence* of an atypical reaction and thus easier to observe and report. Additionally, the literature that specifically focuses on hyper-responsiveness has provided a convincing link between these sensory symptoms and other challenging symptoms (e.g., anxiety, GI problems) that occur in ASD at a disproportional rate. Nonetheless, improved measurement development and analysis techniques will be necessary to enhance the sensitivity of these questionnaires in order to understand individual differences in sensory processing. In addition, the current literature provides some evidence for differences in subjective and observed sensory experiences based on self- and parent- report, respectively. Self-report in ASD has been criticized because verbal and cognitive deficits (e.g., insight) may limit accurate reporting of symptoms (Ozsivadjian et al., 2012); however, these types of questionnaires may be the only way to capture subjective experiences. Parent-report questionnaires in ASD have been criticized for relying on retrospective report, which can lead to inaccurate reporting and recollection biases (Hoyle et al., 2001) and can additionally be influenced by individual factors such as parenting stress (Ooi et al., 2016). However, questionnaires allow for observations across time and contexts and thus may be one of the best ways to evaluate more generalizable observable reactions to sensory input and their impact on day-to-day functioning. Future measure development would benefit from focus on this component rather than attempting to use questionnaires for aspects of sensory processing that cannot be readily observed (e.g., high threshold to stimulation).

### Lab-based observational coding studies

Several lab-based observational coding paradigms have been developed to assess sensory symptoms, including the Sensory Processing Assessment (SPA), Tactile Defensiveness and Discrimination Test—Revised (TDDT-R), Sensory Processing Scales, and Sensory Challenge Protocol. The SPA Baranek et al. (2007) and the TDDT-R were designed as play-based assessments to observe sensory patterns in the lab. The SPA has been validated in children 9 months to 6 years and the TDDT-R has been validated in children 2–14 years; however, these tools are still in development and thus clinical norms have not been published. Additionally, the play-based nature of these assessment tools limits their use to younger and/or lower functioning individuals. The Sensory Processing Scales (Schoen et al., 2014) consists of structured games that involve sensory components (e.g., observe a spinning sparkle wheel; paint your arm with a feather, brush, and rough sponge). Although not developed for individuals with ASD specifically, it has now been applied to high functioning children ages 4–16 years with and without ASD (note: high functioning ASD typically refers to individuals with at least low average cognitive ability). The play-based assessment may also be appropriate for lower functioning individuals, but remains to be tested in this population. The Sensory Challenge Protocol (McIntosh et al., 1999; discussed in the Psychophysiological Studies section) was designed to assess physiological responses to the presentation of items with sensory features (e.g., strobe light for visual, feather touching the face for tactile). This protocol has been used in children ages 4–15 years, but has been modified for use in younger children (McCormick et al., 2014).

Baranek et al. (2007) utilized the SPA, which involves presenting infants and young children with a variety of toys with different sensory features and coding the resulting behaviors. They found increased hyper-responsiveness (sensory aversion) in children with ASD and developmental delay compared to those with TD, and that across all three groups, these symptoms decrease as both chronological and mental age increase. They also found a deficit in sensory orienting in ASD at a mental age of 6 months that normalized by a mental age of 5.5 years. Baranek et al. (2013) specifically looked at sensory orienting using the SPA, confirming the early orienting deficit in ASD and extending this to be true for both social and non-social stimuli, and to be related to joint attention. Foss-Feig et al. (2012) utilized the TDDT-R, a structured behavioral observation paradigm, and tactile specific scores on parent report questionnaires revealing specific associations between tactile hypo-responsiveness and both core features of autism: social communication impairments and restricted/repetitive behaviors. Tavassoli et al. (2016) utilized the Sensory Processing Scales in high functioning children with and without ASD ages 4–15. Children with ASD showed significantly greater sensory reactivity compared to controls, and this reactivity was correlated with parent-reported sensory symptoms across the entire sample.

In these studies, questionnaire measures and lab-based observational coding measures were inconsistently correlated with each other, suggesting that unique information can be gleaned from each measure. This is likely due in part to the limitations of each type of measure: observations from lab-based paradigms may not be generalizable to real life settings and questionnaire measures are prone to reporter biases that decrease internal validity. Although additional paradigm development work is needed, observational coding paradigms represent a more structured and objective way to understand observable reactions to the presentation of sensory input.

Several studies have combined questionnaire-based measures with observational coding paradigms to create composite scores for different sensory response patterns. This approach adheres to the multi-method model and allows for a more comprehensive assessment. One study using composite scores found an association between hyper-responsiveness and repetitive behaviors in both ASD and developmental delay (Boyd et al., 2010) and another found an association between hypo-responsiveness and sensory seeking symptoms with various measures of social and communication deficits (Watson et al., 2011), confirming the findings previously highlighted in studies utilizing only questionnaires. Furthermore, in a study looking at associations between sensory responding patterns and temperament, Brock et al. (2012) found that increased sensory symptoms across all three patterns (hyper-responsiveness, hypo-responsiveness, sensory seeking) were associated with increased withdrawal and negative mood. This multi-method approach is commendable, but future studies should expand on this approach to investigate both shared and unique contributions of symptoms assessed using different methods. Furthermore, hypo-responsiveness may be better understood by methods that do not rely on observation and report given that they are defined by the *absence* of a typical response and may never be able to be accurately assessed by questionnaire or other observational methods.

### Psychophysiological studies

Psychophysiological studies focus on the body's response to sensory stimulation, specifically looking at functioning of the ANS. The ANS functions through the sympathetic and parasympathetic branches, which are responsible for fight or flight responses and the regulation of those responses and maintenance of homeostasis, respectively. This method provides objective measures of the individual's bodily responses to sensory input that have been systematically linked to emotional states.

Three studies have used the Sensory Challenge Protocol while measuring ANS responding and have yielded conflicting results. Schoen et al. (2009) assessed children ages 4–15 years with ASD, Sensory Modulation Disorder, and TD, measuring arousal levels with electrodermal activity, a measure of sympathetic activity. Children with ASD had typical habituation patterns, but lower baseline arousal levels and lower reactivity, especially to the first two stimuli within each modality, suggesting that children with ASD may have a reduced ability to initially attend to, and thus process, environmental stimuli. Interestingly, they also found that parent-reported sensory symptoms were not related to the physiological arousal levels, again pointing to a divergence between lab- and questionnaire-based measures. Schaaf et al. (2015) collected cardiac measures associated with sympathetic and parasympathetic activity in children with and without ASD ages 6–9 years. Respiratory sinus arrhythmia, the measure of parasympathetic activity, was lower in ASD in response to sensory stimulation. However, pre-ejection period, the measure of sympathetic activity, did not differ between groups. These findings suggest that parasympathetic regulatory functions are specifically faulty in children with ASD. Finally, Lane et al. (2012) tested the mediating role of reactivity to sensory stimuli in the path from baseline reactivity to anxiety symptoms in children ages 6–10 years with ASD, attention-deficit hyperactivity disorder, or TD. Findings suggest a direct relationship between parent-reported sensory hyper-responsiveness and child-reported anxiety. Additionally, they found a relationship between baseline reactivity and anxiety fully mediated by reactivity to sensory stimuli, and a relationship between baseline reactivity and habituation partially mediated by reactivity to sensory stimuli. Together, these findings suggest that the initial state of arousal influences the degree of physiological response to sensation, which then determines both the nervous system's recovery ability and symptoms of anxiety. One additional study used a modified version of the Sensory Challenge Protocol in children 2–5 years with and without ASD and found no group differences in psychophysiological responses to sensory stimuli nor relationships between these responses and parent-reported sensory symptoms (McCormick et al., 2014). In sum, the Sensory Challenge Protocol represents an ecologically valid paradigm (presenting real world objects with sensory features), and when combined with psychophysiological measurement, provides objective information about an individual's emotional response beyond the level of behavioral observations. Additional studies using this paradigm are necessary to better understand the currently conflicting results of sympathetic and parasympathetic differences in response to sensory input in ASD.

Two additional studies have tried to relate ANS responses to reported and/or coded observable responses to sensory input. Woodard et al. (2012) presented sensory stimuli to a small group of children ages 2–3 years with and without ASD. They recorded heart rate during the presentations and coded observable reactions. Although heart rate and behavioral codes were only weakly correlated, both measures showed that toddlers with ASD were more hyper-responsive compared to toddlers with TD. Additionally, there was only one significant relationship between scores on the Infant-Toddler Sensory Profile (parent report questionnaire) and heart rate: hypo-responsiveness was negatively associated with heart rate. These findings suggest that questionnaire measures and behavioral ratings are at best a weak indicator of autonomic activity and highlight the importance of including multiple measures to examine sensory processing in ASD. In a study of 5–19 year olds looking at ANS function in ASD and its relation to sensory symptoms, Daluwatte et al. (2015) found that lower pupil constriction amplitude, a measure associated with parasympathetic activity, was related to increased overall sensory symptoms in ASD. The authors conclude that certain atypical sensory symptoms in ASD seem to be related to reduced parasympathetic modulation.

Overall, these studies provide mixed evidence at younger ages and consistent evidence at older ages of ANS dysfunction in ASD, particularly in response to sensory stimulation, with some studies pointing to deficits in sympathetic activity and others pointing to specific deficits in the regulatory role of the parasympathetic system. These studies begin to characterize the body's response to sensory input by using measures that tap the peripheral nervous system. In this way, this collection of studies begins to bridge the neural and symptom literatures, while including an objective measure of the affective component associated with symptoms.

### Summary of sensory symptoms literature

In sum, our understanding of sensory symptoms in ASD has improved somewhat in the past decade. Specifically, larger, better-characterized, and narrower age range samples have provided opportunities to use more advanced analytic approaches and to characterize the development of sensory symptoms. Additionally, the specific focus on hyper-responsiveness taken by several studies has provided the start to a more comprehensive understanding in that pattern of sensory processing. Similarly, development of more specific questionnaires and lab-based paradigms has advanced the measurement tools available and begun to allow for a multi-method approach (e.g., ability to create composite scores using questionnaire *and* observational methods). Finally, an increased focus on psychophysiological responses provides a new level of understanding about the body's underlying responses to sensory stimulation.

## Neural response to sensory input

The sensory symptoms literature presented above focuses on the individual's response to sensory input and highlights the significant impact of these symptoms on individuals with ASD and their families. The neuroscience literature that follows focuses on the brain's *functional* response to sensory input. Thus, studies of brain structure and connectivity patterns, both of which are impacted in ASD and likely contribute to the functional responding differences, are not evaluated here. However, the relevant functional consequences of these differences will still likely be captured.

By in large, neuroscience studies focus on a specific sensory modality and employ a specific methodology based on which characteristics of neural processing are of interest. The two most commonly used methods in ASD research are electroencephalography (EEG) and functional magnetic resonance imaging (fMRI), with EEG studies focusing primarily on the timing of neural responses and fMRI studies focusing primarily on the location of neural responses. Magnetoencephalography (MEG), which captures both timing and location, has also more recently been utilized in ASD. These studies will be reviewed by methodology used and by sensory modality studied.

### Electroencephalography (EEG)

Several studies use EEG to measure cortical reactivity to sensory stimulation, focusing largely on individual components that correspond to specific sensory events (event related potentials; ERP). EEG is a relatively non-invasive approach that can be used successfully across all ages and functioning levels. Task demands are typically minimal and paradigms can be short. Finally, EEG provides high temporal resolution at the expense of spatial resolution. Given this strength of temporal resolution, the following EEG studies highlight both bottom-up and top-down influences on sensory processing, which typically occur at earlier and later temporal stages of processing, respectively. Bottom-up refers to the influence of purely sensory information on processing, while top-down refers to higher-order influences (e.g., attention) that interact with the incoming sensory information.

#### Auditory

The vast majority of EEG studies have targeted the auditory modality. Generally, two paradigms have been used in conjunction with EEG recordings: paired-clicks and auditory oddball tasks. In paired-clicks paradigms, two auditory stimuli are presented in succession allowing for a comparison in cortical response to the first and second (repetitive) input. Typically, there is a decrease in amplitude of these early components of the auditory ERP to a repeated stimulus, reflecting inhibition of the repetitive input, known as sensory gating. Oddball tasks typically utilize three stimuli: a standard stimulus that is the most commonly presented stimulus, a deviant stimulus that is presented less frequently and varies in one dimension (e.g., frequency) from the standard stimulus, and a novel stimulus that is also presented less frequently but varies greatly from the standard stimulus. Typically, there are distinct cortical responses to the deviant and novel stimuli reflecting appropriate change and novelty detection.

Orekhova and Stroganova (2014) recently reviewed the auditory ERP studies in ASD, which include participants across a range of cognitive ability. Early auditory components include the P50 and P100. Studies of sensory gating in ASD collectively show intact P50 responses, but reduced reactivity of the P100 in the right hemisphere. The later auditory ERP components, MMN and P3a, are both involved in processing change events, with MMN being critical for initial deviancy detection and P3a being important for involuntary attention orienting and evaluation preceding a response. The results for these later ERP components in ASD are not entirely consistent, but overall, suggest intact cortical change detection and evaluation when stimuli are within the focus of attention, but show cortical hypo-responsiveness when stimuli are outside the focus of attention, highlighting the importance of top-down modulation of sensory processing in ASD. Additionally, the authors suggest that age and cognitive ability of participant samples may explain some of the discrepant findings across studies.

Donkers et al. (2015) sought to relate auditory ERPs to sensory symptom patterns in children with ASD ages 4–12 across a range of cognitive ability. Utilizing an auditory oddball paradigm, they found marginally smaller amplitudes of the early P1 and N2 ERPs to standard tones, smaller P3a amplitude to novel tones, and longer P1 latency to deviant tones in ASD compared to TD. Although no single ERP component predicted any sensory symptom pattern, complex and conditional associations between auditory ERPs and sensory symptom patterns were revealed, highlighting both bottom-up (early sensory) and top-down (attentional) influences on the severity of sensory symptoms in ASD. Sensory symptoms were assessed broadly (i.e., across all sensory modalities) and included both parent report and behavioral observation measures to calculate composite scores of sensory hyper-responsiveness, hypo-responsiveness, and seeking. This attempt to relate neural responsiveness to symptoms is commendable; however, the neural measure was exclusively in the auditory modality while the symptoms were more broadly assessed across multiple sensory modalities.

#### Visual

Whereas the majority of EEG studies have been in the auditory modality, an increasing number of studies have used visual paradigms. Findings regarding early visual processing are mixed. Individuals with ASD across a range of cognitive ability showed hyper-responsiveness to flashes of light, demonstrated by stronger and quicker initial visual evoked responses and a slower recovery (Isler et al., 2010). In a study investigating cortical representation of the visual periphery in ASD, individuals with high functioning ASD had larger early responses (increased P1 amplitude) to checkerboard pattern stimuli presented in the periphery (Frey et al., 2013), suggesting early hyper-responsiveness and increased cortical representation of the visual periphery in ASD. Visual evoked responses to grating stimuli suggest a dissociation between hyper- and hypo-responsiveness based on spatial frequency, with high and low spatial frequency of a stimulus yielding increased and decreased responding, respectively (Vlamings et al., 2010; Pei et al., 2014). Hypo-responsiveness may be further restricted to the right hemisphere (Pei et al., 2014). Two studies investigated later components of visual processing by using visual oddball tasks in children with and without ASD. Both found cortical hyper-responsiveness to visual change, regardless of whether it was an active (Baruth et al., 2010) task with high functioning ASD or a passive (Clery et al., 2013b) task with individuals with ASD across a range of cognitive ability. These results suggest that attention to visual input does not differentiate hyper- and hypo-responsiveness in the same way that it seems to in the auditory modality.

Milne et al. (2009) studied visual processing by examining changes in EEG power, which provides an index of neural synchronization. Participants with and without ASD across a range of cognitive ability viewed grating stimuli of varying spatial frequencies and were asked to press a button each time they saw a zebra on a screen. Overall, they found earlier peak latencies for the early C1 and P1 ERPs in ASD supporting faster reactions to basic visual stimuli. After localizing the clusters that accounted for the greatest amount of variance in EEG activity, the authors found stronger power in ASD in cingulate gyrus (reflecting greater attentional control), reduced effect of spatial frequency in ASD in striate and extrastriate regions (reflecting less neural specialization in networks recruited for basic visual perception), and no differences in the parietal region. Together, these findings suggest that early visual areas (e.g., primary visual cortex) are not hyper-responsive in ASD, but rather that there is reduced modulation of the networks involved in basic visual perception that likely contributes to the disruption of perceptual binding in ASD. There is also evidence of reduced synchrony of visual areas between the right and left hemisphere (Isler et al., 2010).

#### Tactile

Only one study has used ERP to investigate the brain's functional response to non-social tactile stimulation in high functioning children and adolescents with ASD, delivering air puffs to participants' finger tips while they were attending to this stimulation (Cascio et al., 2015). Although no group differences were seen in the ERP response to the stimulation, ERP responses at different time points post-stimulus were related to tactile hyper- and hypo- responsiveness as measured by parent report. These findings suggest that earlier neural responses to tactile stimulation are related to tactile hyper-responsiveness, while slightly later neural responses are related to tactile hypo-responsiveness and may involve higher-level processes such as attention allocation and assignment of emotional valence.

In sum, the EEG literature has revealed important differences in the timing of response to auditory, visual, and tactile input in ASD. Although many studies suggest atypicalities in temporally later stages of sensory processing, earlier stages have also been implicated. Additionally, both bottom-up and top-down processes seem to be affected, with top-down processes (e.g., attention) possibly differentiating impairment, at least in the auditory modality. Importantly, each sensory modality provides slightly different conclusions, providing merit to the single sensory modality approach in the study of neural mechanisms of sensory processing. Finally, existing attempts to relate EEG measures to questionnaire measures serve as models for integrating neural and symptom perspectives.

### Functional magnetic resonance imaging (fMRI)

Rather than measuring the electrical activity of neurons like in EEG methods, fMRI is based on the indirect measurement of brain activity through changes in blood flow. Predicated on the assumption that increased blood flow (i.e., activation) to a neural region is indicative of increased neural activity, fMRI studies provide exceptional spatial resolution at the expense of temporal resolution. Unlike EEG, which has been utilized across a variety of ages and functioning levels, fMRI is typically used in older and higher functioning ASD participants, who can remain still for the duration of the paradigm and tolerate the MRI environment. The studies described below use high functioning samples and often include participants that span a broad age range. Although the majority of the fMRI studies in ASD investigate higher order cognitions (e.g., theory of mind, face processing, language processing), several studies, outlined below, assess basic sensory processing. These studies provide information about where in the brain sensory processing differences exist in ASD. These findings largely parallel the EEG findings, in that regions involved in purely sensory processing and those involved in attentional control both show atypical responding patterns in ASD.

#### Auditory

Gomot et al. (2006, 2008) demonstrated the modulating role of attention across two auditory oddball fMRI studies, one with a passive listening task and one with an active task. Children and adolescents with ASD showed lower brain activation in the passive task and higher brain activation in an active task, in both parietal and frontal areas in response to deviant and novel stimuli. These results show both neural hypo-responsiveness (Gomot et al., 2006) and hyper-responsiveness (Gomot et al., 2008) to auditory change dependent on attention, consistent with the ERP data of the MMN and P3a components (Orekhova and Stroganova, 2014). Thus, the role of attention seems to be critical in auditory change perception in ASD, with consistent findings across EEG and fMRI studies. In a study of adolescents and adults with ASD investigating non-social auditory complexity, temporal but not spatial stimulus complexity was associated with increased and decreased fMRI activity in ASD in primary and other (anterolateral and posterior superior temporal gyrus) auditory cortical regions, respectively (Samson et al., 2011).

#### Visual

In a small fMRI visual attention study looking at children with ASD, their unaffected siblings, and controls (Belmonte et al., 2010), children with ASD had decreased activation in attention networks during the trials, but they and their unaffected siblings showed delayed activation of these networks (immediately following each trial). The unaffected siblings showed greater delayed attention network activation suggesting a stronger compensatory process that may be protective against developing symptoms of ASD. Clery et al. (2013a) looked at visual change detection using fMRI in a small group of adults with and without ASD. Adults with ASD showed increased activation in visual areas and decreased activation in frontal areas to deviant and novel stimuli, consistent with the idea of increased sensory processing and reduced top-down modulation of sensory areas. The anterior cingulate cortex, a region important for attention switching and allocation of attentional resources, was also shown to be hyper-responsive in ASD while processing visual change events, offering an explanation of attention switching deficits as a mechanism for altered visual change detection in ASD. Thus, auditory change detection seems to be dependent on attention engagement, and visual change detection may be dependent on attention switching.

Ohta et al. (2012) investigated sensory (visual) filtering by implementing an fMRI paradigm manipulating perceptual load and presence of a distractor stimulus in adults with and without ASD. In typical individuals, the degree of processing of unattended stimuli is dependent on task load (e.g., greater processing when task load is low), which reflects efficient processing abilities. Results from this study showed no group differences in activation in the fronto-parietal attention network across conditions, but found that distractor-evoked activity in visual cortex was modulated less by perceptual load in ASD. These findings suggest a lack of flexible top-down regulation of sensory processing.

#### Tactile

Two fMRI studies have looked at responses to tactile stimulation in ASD. Kaiser et al. (2016) investigated neural responses to touch on the palm in children and adolescents with and without ASD, and found increased response in primary somatosensory cortex and insula in ASD, suggesting hyper-responsiveness to non-social touch. Cascio et al. (2012) demonstrated increased fMRI activation in attention areas in adults with ASD when presented with aversive, but not pleasant, tactile stimulation. This study also collected subjective ratings of roughness and pleasantness for the same stimuli and found no correlations between these ratings and the fMRI response, highlighting a disconnect between neural responding and subjective experiences. Together, these studies suggest neural hyper-responsiveness to basic tactile stimulation, but there is inconsistency as to whether this hyper-responsiveness is localized in sensory (Kaiser et al., 2016) or attention (Cascio et al., 2012) areas.

#### Multiple modalities

Two fMRI studies have made notable attempts to link neural responding to sensory symptoms by investigating sensory and limbic responses to mildly aversive sensory stimuli (Green et al., 2013: auditory, visual, and audiovisual combined stimuli; Green et al., 2015: auditory, tactile, and audiotactile combined stimuli) in children and adolescents with and without ASD. Across both studies, they found neural hyper-responsiveness in ASD across the unisensory and multisensory conditions in both sensory and limbic areas, including frontal regions, with the strongest increases in neural responding during the multisensory condition. In the multisensory conditions, signal increase in several areas, including sensory, limbic, and frontal regions, was positively associated with sensory hyper-responsiveness scores on a composite variable generated from two parent report questionnaires (Short Sensory Profile and SensOR) above and beyond anxiety levels. Unfortunately, a similar analysis was not reported in the unisensory conditions. Green et al. (2015) conducted additional analyses looking at the specific role of sensory hyper-responsiveness symptoms, finding that those with ASD *and* high sensory hyper-responsiveness symptoms were driving the effects of neural hyper-responsiveness, and that those with ASD only (and low sensory hyper-responsiveness symptoms) were similar to controls. Connectivity analyses suggest that the hyper-responsiveness in primary sensory areas observed in those with ASD and high hyper-responsive symptoms may lead to hyper-responsiveness in limbic areas, which may then over-engage frontal areas in an attempt to regulate the response. Together, these two studies highlight the value of investigating differences in neural responding patterns based on sensory subgroups within ASD, the role of top-down modulation, and the value of combining neural and symptom measures.

In sum, there are many fewer fMRI studies investigating neural response patterns to basic sensory stimuli compared to the EEG literature. However, the few studies outlined above allow for preliminary understanding of how the brain responds when processing basic auditory, visual, and tactile stimuli. In higher order regions, atypical responding patterns are consistently reported in ASD; however, the evidence is mixed in that some reports show hyper-responsiveness and other reports show hypo-responsiveness. In purely sensory regions, atypicalities in ASD are not always observed; however, when these atypicalities are observed, evidence is overwhelmingly in the direction of neural hyper-responsiveness, especially in the most simplistic tasks (e.g., presenting sensory stimuli with no requirement of task engagement). Recent advancements in this literature highlight the value of integrating neural and symptom perspectives.

### Variability in responding to sensory input in EEG and fMRI studies

The studies reviewed above focus on the amount of responding to a particular sensory stimulus collapsed across several stimulus presentations. Another approach is to look at the variability in responding across the different stimulus presentations to determine the consistency or amount of “noise” in the neural response. In fact, increased (or decreased; Davis and Plaisted-Grant, 2015) neural noise as a heuristic theory of ASD has generated a lot of attention recently (Dinstein et al., 2015). Four neural studies provide empirical evidence for increased variability in neural responding to basic sensory input, consistent with such an account. Milne (2011) reanalyzed a subset of the data from Milne et al. (2009) to investigate intra-participant variability of the P1 (early sensory) cortical response (amplitude and latency) and inter-trial phase coherence. Inter-trial phase coherence measures the degree to which EEG activity is phase-locked to a specific stimulus presentation across trials. They found increased intra-participant variability in the children with ASD across all three measures, pointing to evidence for increased neural noise in ASD. Weinger et al. (2014) also reported increased neural noise in a sample of children with ASD, as measured by EEG in the visual modality. They presented checkerboard pattern stimuli and found no group differences in amount of neural responding; however, they found decreased signal to noise ratio and increased neural noise (greater variability in amplitude across trials) in ASD.

Similar findings of increased neural noise exist across two fMRI studies (Dinstein et al., 2012; Haigh et al., 2015) in adults with ASD. Visual, auditory, or tactile stimuli were presented while participants completed an unrelated task to divert attention away from sensory input. There were no differences in amount or location of neural activation to these stimuli; however, the variance of responses was larger in ASD compared to controls. This increased variance was specific to sensory regions during stimulus presentation; it was not seen in other neural areas during stimulus presentation or in sensory areas during rest. Overall, these studies of variability in neural response do not support theories of neural hyper- or hypo-responsiveness to simple sensory input (at least when attention is diverted away), but offer increased intra-participant neural variability as a consistent marker of sensory processing dysfunction in ASD.

### Magnetoencephalography (MEG)

Three studies of sensory processing in ASD have used MEG, two in the auditory and one in the tactile modality. MEG provides similar temporal resolution to EEG but with better spatial resolution. Roberts et al. (2010) conducted an MEG study on auditory processing in high functioning children with and without ASD and found delayed M100 latency in ASD across all four tone frequencies presented. There were no group differences in the M50 response. Typical maturational processes of the M100 response becoming earlier with age was observed in the control group, but absent in the ASD group, suggesting atypical maturation of the auditory cortex in ASD. Orekhova et al. (2012) utilized a paired clicks paradigm (detailed above in the EEG section) with MEG in children with and without ASD, and found atypical P100 lateralization in children with ASD. Children with ASD showed less right lateralization in response to the auditory stimulus, and this was correlated with total sensory problems measured by a parent questionnaire. The child P100 is thought to be involved in arousal, spatial orienting, and attention processing, all of which are typically right lateralized functions. Atypical P100 lateralization seen in ASD may reflect disrupted preattentive arousal, in which children with ASD rely on non-optimal left hemisphere processing.

In a tactile MEG study with high functioning children with and without ASD, Marco et al. (2012) applied a finger tapping paradigm to investigate the timing and amplitude of responses in primary somatosensory cortex. Children with ASD had reduced responses in the slow, but not fast, rate version of the paradigm, specifically in the left hemisphere. Additionally, tactile sensitivity scores on the Sensory Profile correlated with amount of neural response in primary somatosensory cortex across the combined sample. An additional analysis separating the groups by tactile sensitivity scores revealed more robust neural differences between these groups in both the right and left hemisphere, suggesting that these neural differences are more closely related to individual differences in tactile functioning than to ASD specifically. Together, these three studies point to maturational and lateralization differences related to sensory processing that may be present in ASD, and indicates the need for further work using this approach.

### Summary of neuroscience literature

In sum, the neural processing of sensory input in ASD literature points to atypical neural processing of even the most basic sensory stimuli that can be observed across a variety of methodologies. EEG studies provide evidence for atypicalities in ASD during both earlier and later sensory processing stages. fMRI studies provide evidence for different spatial activation patterns across areas of the brain responsible for these earlier and later sensory processing stages. Emerging MEG evidence points to maturational and lateralization differences related to sensory processing that may be present in ASD. Higher-order behaviors at the symptom level are complex and arise from complicated interactions of these simpler processes. Although we are far from understanding these complicated interactions, this neural literature highlights the basic nature of some of the differences associated with ASD. Additional research building upon existing efforts to combine neural and symptom perspectives is necessary to sort through the broad array of findings outlined above.

## Psychophysics: an intermediate approach

Psychophysics is an approach that has been recently applied to the study of sensory processing in ASD by both neuroscientists and sensory symptom researchers. Psychophysical studies rely on a decision related to a perceptual experience, and are designed to closely model neural responding patterns. Thus, this approach capitalizes on an intuitive intermediary between the neural response to sensory input and the individual's observable reaction. Additionally, psychophysical studies allow for the study of isolated features of real world stimuli that can be conceptualized as the building blocks of higher-level perception. For example, if studying motion perception within the visual modality, one would present the most basic motion stimulus (i.e., a moving grating pattern) and determine an individual's ability to perceive that stimulus in either a detection task (e.g., press a button when you see the moving stimulus on the screen) or a discrimination task (e.g., decide if the stimulus is moving to the right or the left). Although a review of this literature is beyond the scope of this paper, examples of this approach applied to ASD include multiple sensory modalities, including visual (e.g., Bertone et al., 2005), auditory (e.g., Jones et al., 2009), and tactile (e.g., Cascio et al., 2008).

Although these types of measurements lie in between neural response and observable reactions, this method on its own has yet to provide an integrative framework for understanding sensory processing in ASD. This is likely because important links are missing between neural responding, detection/discrimination decisions, and observable reactions. In fact, a large research area in the field of basic neuroscience seeks to understand how neural firing translates to these types of decisions in humans generally (and primates more globally). Additionally, much remains to be known about the link between these more basic perceptual decisions and higher-order observable reactions. In particular, these measurements largely ignore the affective component and real-life impact that characterize sensory symptoms of ASD. However, these tools may allow us to characterize hypo-responsive symptoms in a way that questionnaires and observational coding paradigms miss, because this category of symptoms is defined by the absence of typical reactions.

## Recommendations for future research

Until 2005, sensory processing research in ASD was largely focused on whether individuals with ASD exhibited atypical sensory responses and if these sensory responding patterns could be used to differentiate ASD from other developmental disorders (e.g., intellectual disability, Fragile X syndrome). Because of these motivations, sensory processing research was mostly descriptive and one-dimensional. Additionally, this early research suffered from studies with small, and often poorly characterized samples resulting in uncertainty about the presence and uniqueness of sensory processing difficulties in ASD. In the last decade, sensory processing research in ASD has expanded significantly—in the number of studies published, increased methodological rigor of these studies, and diversification of approaches used. This has led to a *multidisciplinary* understanding of sensory processing in ASD. Currently, the field has a vast amount of descriptive and emerging mechanistic information about how individuals with ASD (and possible subgroups within ASD) perceive and respond to sensory information differently. However, this information has been generated from two very different perspectives: clinical science and neuroscience. In order to integrate this information into a cohesive picture of *how* and *why* sensory processing differences manifest in ASD and to be able to see the translational value in its application to early identification and treatment, it is essential for these two perspectives to communicate more effectively and move toward an *interdisciplinary* understanding. Specific recommendations are outlined below that will hopefully allow us to embark on the next phase of sensory processing research in ASD.

### What can sensory symptom researchers learn from neuroscientists?

#### Greater use of modality-specific measurement

One of the difficulties with the current approach to studying sensory symptoms in ASD is the use of measures that collapse across auditory, visual, tactile, and other sensory modalities. The neuroscience research has revealed important differences between sensory processing modalities in ASD and has highlighted the importance of precision and specificity in conceptualizing atypical responses. The sensory symptom literature has begun to address this by using subscale or factor scores that isolate specific modalities. However, the use of more modality-specific measures, both questionnaire and lab-based, will improve sensitivity of these measures and hopefully help begin to bridge knowledge from the neuroscience and clinical perspectives.

#### Appropriate selection of measurement based on pattern of responding

In line with the above recommendation, the most appropriate measurement tool may depend on the pattern of responding of interest. For example, hyper-responsiveness may be best investigated with questionnaires and lab-based observational coding paradigms, if these measurement tools are enhanced. However, hypo-responsiveness is difficult to capture with observation in the lab or via parent questionnaires, given that these symptoms are defined as the *absence* of typical responding. Such symptoms may be more directly and accurately captured using psychophysical approaches that rely on detection and/or discrimination thresholds (i.e., the minimum amount of sensory input needed for reliable perception).

### What can neuroscientists learn from sensory symptoms researchers?

#### Increased focus on developmental change over time

The sensory symptoms literature has moved toward investigating specific developmental periods, with a few longitudinal studies that directly explore developmental effects, but the neuroscience research lags behind in this manner. Although the symptoms literature suggests little developmental change at the symptom level in childhood, developmental differences do seem apparent when one looks across the lifespan. Future neuroscience studies should consider narrower age ranges and specific consideration of developmental changes occurring at those ages, as developmental changes may be more pronounced at the neural level. Although there are some longitudinal studies investigating structural neural changes in ASD, longitudinal studies examining functional responding to sensory input are lacking.

#### Recognition and investigation of the heterogeneity within sensory processing in ASD

The sensory symptom literature provides emerging evidence for sensory-based subgroups in ASD. It will be beneficial for neuroscientists to appreciate the heterogeneity within ASD, rather than conceptualizing ASD as a single, homogenous disorder. Given the sensitivity and precision of neuroscience approaches, it is possible that these approaches will aid our understanding of this heterogeneity and reveal meaningful subgroups within the disorder.

#### Greater emphasis on top-down modulation of sensory processing

From the sensory symptom literature, the modulation of sensory input is most related to clinical problems. The current neural literature also points to the importance of the modulatory role of cognitive processes, such as attention, on sensory processing. Thus, future studies should continue to examine these top-down effects in an effort to better map the mechanisms involved in the presentation of sensory symptoms in ASD.

### General recommendations

#### Exploration of sensory processing in lower functioning individuals with ASD

The sensory symptoms literature suggests that sensory difficulties are present across functioning levels in ASD. However, the presentation of these difficulties likely differs across various levels of analysis. Current measures, particularly those with the greatest sensitivity, are largely limited to high functioning individuals with ASD, thus over-representing this population in research. Although measure development for lower functioning individuals is challenging, adaptation of measures for this population presents an opportunity for creative collaboration between fields.

#### Increased understanding that the relationship between neural hyper- and hypo-responsiveness and symptoms of hyper- and hypo-responsiveness is highly complex

It is tempting to assume that the relationship between neural responding and observable reactions is simplistic, and many of the current theories about sensory functioning in ASD adopt that framework. This is especially true given the shared terminology used to describe both responses. However, this assumption leads to oversimplification, which hinders progress toward unraveling the complex reality of these relationships. Each field is able to provide information on specific aspects of sensory processing, but researchers should be mindful of the limitations of each method.

#### Utilization of a multi-method approach to assess different aspects of sensory symptoms

Several studies that have used multi-method approaches suggest divergence among different aspects of sensory processing. In fact, review of the literature suggests that each method contributes to our understanding of sensory processing in a unique way. Questionnaires and lab-based observational coding paradigms can be best used to characterize observable reactions to sensory input, most notably in the hyper-responsive pattern, and the day-to-day impact on functioning; psychophysiological measurements add additional information about the underlying bodily response, including an emotional reaction component; psychophysical methods characterize the perceptual responses (detection/discrimination) that result from atypical neural processing; and direct neural measures (EEG, fMRI, MEG) provide the best information about underlying timing, degree, and location of neural response to sensory input. An important caveat to consider is that each of these methods is only as useful as the measurement tool selected, and measurement work, particularly for questionnaires, continues to be necessary. However, by improving upon and then combining these methodologies, we will protect against over-interpretation of any single-method. Specifically, each method can contribute knowledge about a specific and appropriate *aspect* of sensory processing rather than attempt to make claims about sensory processing as a whole. Then, upon combination of these different methods, a more accurate understanding of sensory processing in ASD can be achieved.

## Conclusions

The interest in sensory processing in ASD has expanded substantially in the last decade, as evidenced by an increased number of studies using well-characterized samples with sufficient sample sizes, the development of new measures and paradigms, and the adoption of neuroscience approaches. At this point, sensory processing should no longer be conceptualized as a single construct that can be measured similarly by different tools. Instead, each approach offers a unique contribution to a piece of sensory processing, and if applied appropriately, the understanding of sensory processing in ASD as a whole can progress. It is our hope that this paper highlighted the importance of sensory processing in ASD, explained the two major research perspectives related to sensory processing in ASD, and provided a framework for conceptualization of sensory processing moving forward. Finally, by first understanding the link between brain and symptoms *within* the sensory domain, we can more successfully understand the relationship between brain and symptoms in ASD more broadly.

## Author contributions

KS conceived the idea and drafted the first version of the paper. LB supervised the manuscript and reviewed the paper for intellectual content. KS and LB revised the manuscript. Both authors approved the final version.

## Funding

KS and LB were supported in part by R01 DC009439.

### Conflict of interest statement

The authors declare that the research was conducted in the absence of any commercial or financial relationships that could be construed as a potential conflict of interest.

## Footnotes

^1^Hyper-responsive and over-responsive are synonymous terms, as are hypo-responsive and under-responsive. The term hyper-responsive and hypo-responsive will be used exclusively in this paper.
